# Correlation of positive RT-PCR for tyrosinase in peripheral blood of malignant melanoma patients with clinical stage, survival and other risk factors

**DOI:** 10.1054/bjoc.1998.0887

**Published:** 1999-12-08

**Authors:** T M Proebstle, W Jiang, J Högel, U Keilholz, L Weber, C Voit

**Affiliations:** Department of Dermatology, University of Mainz, Mainz, Germany; Department of Dermatology, Department of Biometry and Medical Documentation, University of Ulm, Ulm, Germany; Department of Medicine III, University Hospital Benjamin Franklin, Free University of Berlin, Berlin, Germany; Department of Dermatology, Charité, Humboldt University Berlin, Schumannstr. 20/21, Berlin, 10117, Germany

**Keywords:** circulating tumour cells, minimal residual disease

## Abstract

The clinical value of the reverse transcription polymerase chain reaction (RT-PCR) assay for tyrosinase in peripheral blood of melanoma patients is still under debate. A total of 212 blood samples from 212 melanoma patients in all clinical stages (AJCC) were examined. Erythrocytes were lysed prior to RNA extraction by phenol precipitation from 2.7 ml of blood. cDNA for tyrosinase PCR was synthesized using random hexamers. Positive tyrosinase RT-PCR results were obtained in 11% of 106 stage I patients, 18% of 56 stage II patients, 31% of 26 stage III patients and 67% of 24 stage IV patients. After a median follow-up of 36 months (range 26–41), stage III patients with positive RT-PCR for tyrosinase had a shortened disease-free interval as compared to negative patients (*P*< 0.01). In stage IV patients, median overall survival was 8 months in case of a positive RT-PCR in contrast to 12 months in case of a negative test. While univariate analysis showed sex and primary tumour location associated with positive RT-PCR, multiple regression analysis revealed clinical stage and detection of tyrosinase transcripts in peripheral blood as best prognostic factors. Hazard ratios for disease-free survival were 19.7 (confidence interval (CI) 8.53–45.5, *P* = 0.0001) for metastatic vs primary disease and 2.96 (Cl 1.49–5.89, *P* = 0.002) for positive vs negative tyrosinase RT-PCR. The corresponding hazard ratios for overall survival were 97.0 (Cl 12.7–741, *P* = 0.0001) and 4.33 (Cl 1.69–11.1, *P* = 0.002). Our results emphasize the importance of tyrosinase RT-PCR testing in peripheral blood. © 2000 Cancer Research Campaign


*British Journal of Cancer* (2000) **82**(1), 118–123
© 2000 Cancer Research Campaign
Article no. bjoc.1999.0887

### Correlation of positive RT-PCR for tyrosinase in peripheral blood of malignant melanoma patients with clinical stage, survival and other risk factors

**TM Proebstle[1], W Jiang[2], J Högel[3], U Keilholz[4], L Weber[2] and C Voit[5]**

[1]Department of Dermatology, University of Mainz, Mainz, Germany; [2]Department of Dermatology and [3]Department of Biometry and Medical Documentation, University of Ulm, Ulm, Germany; [4]Department of Medicine III, University Hospital Benjamin Franklin, Free University of Berlin, Berlin, Germany; [5]Department of Dermatology, Charité, Humboldt University Berlin, Schumannstr. 20/21, 10117 Berlin, Germany

**Summary** The clinical value of the reverse transcription polymerase chain reaction (RT-PCR) assay for tyrosinase in peripheral blood of melanoma patients is still under debate. A total of 212 blood samples from 212 melanoma patients in all clinical stages (AJCC) were examined. Erythrocytes were lysed prior to RNA extraction by phenol precipitation from 2.7 ml of blood. cDNA for tyrosinase PCR was synthesized using random hexamers. Positive tyrosinase RT-PCR results were obtained in 11% of 106 stage I patients, 18% of 56 stage II patients, 31% of 26 stage III patients and 67% of 24 stage IV patients. After a median follow-up of 36 months (range 26–41), stage III patients with positive RT-PCR for tyrosinase had a shortened disease-free interval as compared to negative patients ($P < 0.01$). In stage IV patients, median overall survival was 8 months in case of a positive RT-PCR in contrast to 12 months in case of a negative test. While univariate analysis showed sex and primary tumour location associated with positive RT-PCR, multiple regression analysis revealed clinical stage and detection of tyrosinase transcripts in peripheral blood as best prognostic factors. Hazard ratios for disease-free survival were 19.7 (confidence interval (CI) 8.53–45.5, $P = 0.0001$) for metastatic vs primary disease and 2.96 (Cl 1.49–5.89, $P = 0.002$) for positive vs negative tyrosinase RT-PCR. The corresponding hazard ratios for overall survival were 97.0 (Cl 12.7–741, $P = 0.0001$) and 4.33 (Cl 1.69–11.1, $P = 0.002$). Our results emphasize the importance of tyrosinase RT-PCR testing in peripheral blood. © 2000 Cancer Research Campaign

**Keywords:** circulating tumour cells; minimal residual disease

The incidence of cutaneous melanoma in Germany – as in many other parts of the world – has increased steadily throughout the past decades. Presuming an incidence of 15 melanomas per 100 000 inhabitants, around 12 000 new cases will be expected in Germany in 1998 and more than 2000 of those patients will die within the next 5 years from metastatic disease. Prognostic factors like Breslow's tumour thickness, location of the primary tumour and patient's sex are well established (Balch et al, 1985). However, individual tumour markers for malignant melanoma are lacking so far. Such individual markers would be of particular value in monitoring the therapeutic efficacy of relatively toxic adjuvant treatment schedules (Kirkwood et al, 1996) or could serve as stratification factors in prospective clinical trials.

At present, in haematology as well as in oncology, efforts of many research programmes are focused on the early detection of micrometastases, circulating malignant cells or early cancer relapses. Promising concepts like the detection of tumour-specific gene mutations or tissue-specific transcripts, allowing recognition of one tumour cells within 10 ml of blood (Brossart et al, 1993) or within 10 million of peripheral blood mononuclear cells (PBMCs) (Ghossein et al, 1998) have evolved. Most authors were able to detect one tumour cell in the background of $10^6$ mononucleated cells (Kunter et al, 1996; Mellado et al, 1996; Reinhold et al, 1997). In melanoma disease, tyrosinase, the key enzyme of melanin biosynthesis (Kwon et al, 1987; Ponnazhagen et al, 1994) was the first tissue-specific transcript to be detected in peripheral blood (Smith et al, 1991), bone marrow (Ghossein et al, 1996), draining lymph nodes (Wang et al, 1994; Schwürzer-Voit et al, 1996; Van der Velde et al, 1996) and in the subcutaneous fat adjacent to primary tumours as well (Proebstle et al, 1996). Since the original description of a reverse transcription polymerase chain reaction (RT-PCR) method for detection of tyrosinase transcripts in peripheral blood (Smith et al, 1991), controversial data have been reported in terms of sensitivity of this method and its clinical relevance (Brossart et al, 1993, 1994; Battayani et al, 1995; Foss et al, 1995; Hoon et al, 1995; Pitman et al, 1996; Gläser et al, 1997; Jung et al, 1997; Farthmann et al, 1998; Ghossein et al, 1998). Striking differences have been reported concerning the detected proportion of tyrosinase-positive patients varying, e.g. in stage IV patients, actually from zero (Foss et al, 1995) to 100% (Brossart et al, 1993). Disease-free and overall survival in correlation with RT-PCR tests have so far been rarely addressed (Kunter et al, 1996; Mellado et al, 1996; Ghossein et al, 1998).

In this study, we examined 212 blood samples from 212 melanoma patients with all clinical stages. Blood samples were obtained as patients presented for routine tumour follow-up. RT-PCR results were associated with clinical tumour stage at the time of blood sampling, furthermore with disease-free and overall survival intervals, as well as with established risk factors like sex and primary tumour location.

*Received 22 October 1998*
*Revised 21 June 1999*
*Accepted 7 July 1999*

*Correspondence to:* C Voit

### MATERIALS AND METHODS

### Patients, staging and processing of blood samples

Samples of 2.7 ml EDTA blood were taken from 212 melanoma patients with all clinical stages. Twenty millilitres of erythrocytelysis (EL) buffer were added within 1 h. The mixture was kept on ice for 15 min and subsequently centrifuged at 1000 rpm for 10 min at 4°C. The pellet was resuspended in 20 ml EL buffer for 5 min on ice, before centrifugation was repeated. One millilitre of guanidinium-iso-thiocyanate (GIT) buffer was added to the pellet. The sample was stored at –85°C until further analysis.

All blood sampling procedures were performed concomitantly to a tumour staging procedure as patients presented for melanoma follow-up. This design was chosen to allow evaluation of the prognostic value of tyrosinase RT-PCR for all-day usage in routine follow-up of melanoma patients. The staging interval and the extent of the staging schedule depended on the current stage of melanoma disease. Routine staging intervals were 12 months for stage IA patients, 6 months for stage IB patients and 3 months for stage II–IV patients. A routine staging procedure included physical examination and ultrasound B-scan of regional soft tissues, detecting more than 90% of the relapses. Ultrasound B-scan of the abdomen and chest X-ray were added once a year for stage I and stage II patients, twice yearly for stage III patients and every 3 months in stage IV patients. Computerized tomography (CT) or magnetic resonance tomography (MRT) scans of the brain, thoracic, abdominal and pelvic organs was performed every 3 months in stage IV patients, at the first clinic visit of stage II–IV patients and whenever a patient had disease progression by tumour stage.

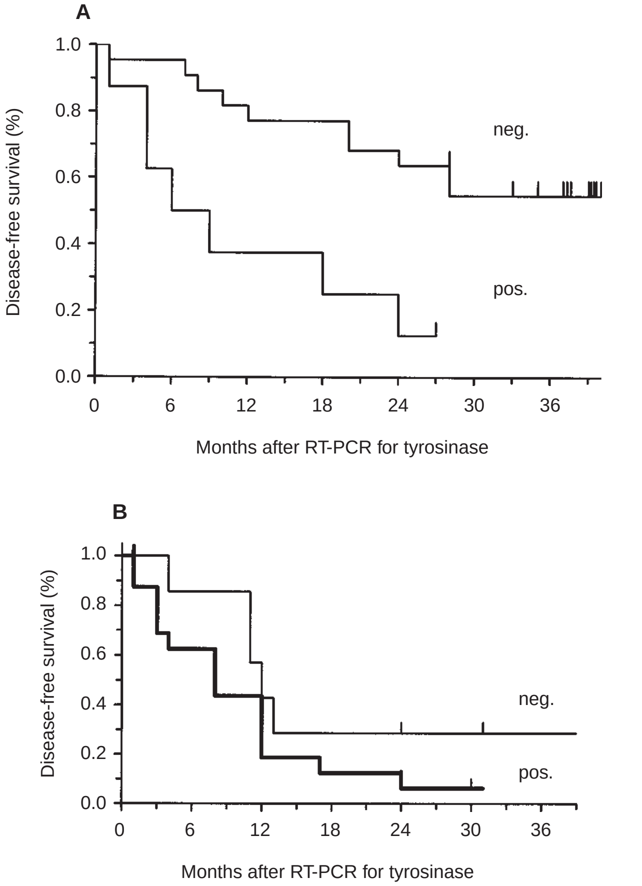

**Figure 1** **(A)** Disease-free survival of stage III patients calculated from the time of blood sampling displayed according to the method of Kaplan and Meier. Log-rank testing revealed statistical significance at a level of $P < 0.01$. The median follow-up interval is 36 months. **(B)** Overall survival of stage IV patients calculated from the time of blood sampling. Differences were not significant ($P = 0.33$). The follow-up intervals of censored patients are tick-marked. One tyrosinase-positive patient died 1 month after blood sampling from pneumonia

### RNA preparation and reverse transcription

Total RNA was isolated according to the method of Chomczynski and Sacchi (1987), and eventually extracted in 60 µl of DEPC water. RNA was analysed at 260 and 280 nm using a spectral photometer. Total yield and purity of the samples were determined before reverse transcription. Isolated RNA was immediately processed or stored at –85°C. Reverse transcription was performed using the superscript preamplification kit (Gibco-BRL). A total of 1.5 µg of RNA were transcribed by means of 50 ng of random hexamers, 10 mM dNTO-mix and 200 units of superscript II RT (Gibco-BRL Inc., Grand Island, NY, USA). The isolated cDNA was further processed or stored at –20°C.

### Nested PCR for tyrosinase

To avoid contamination, reaction mixtures were prepared using a laminar air flow. Primers for tyrosinase-PCR, designed for a nested PCR, were applied as previously described (Smith et al, 1991). The outer primers $HTYR_1$ (TTG GCA GAT TGT CTG TAG CC) and $HTYR_2$ (AGG CAT TGT GCA TGC TGC TT) amplified a product of 284 base pairs. Two microlitres of the previously obtained cDNA sample were added to 5 µl of the tenfold concentrated PCR buffer, 5 µl of 10 mM nucleotides (dATP, DCTP, dTTP, dGTP), 0.4 µl of the primers $HTYR_1$ and $HTYR_2$ (100 pmol $l^{-1}$) and finally aqua bidest was added to a total volume of 50 µl. The PCR-protocol was then initiated on a thermocycler starting with 5 min at 94°C, first hold at 80°C, followed by addition of 1 U (5 U $\mu l^{-1}$) *Taq* polymerase (Gibco). Each of the following 30 cycles involved a first step at 94°C for 45 s, a subsequent step at 60°C for 45 s and a third step at 72°C for 45 s. After 30 cycles a final delay followed by 5 min at a temperature of 72°C and further 5 min at 30°C. One microlitre of this outer PCR product was then diluted 1:100. Five microlitres of this solution were used for the second inner amplification cycle. The nested PCR was carried out in the same way as the primary PCR, inner primers $HTYR_3$ (GTC TTT ATG CAA TGG TT) and $HTYR_4$ (GCT ATC CCA GTA AGT GGA CT) were applied in the same concentration. Ten microlitres of the final product (207 bp) of the second inner PCR were analysed on a 2%-agarose gel (90 V, 80 min). On each gel, length standards (100 bp ladder, Gibco), as well as positive and negative controls were run in parallel to patients' samples (Figure 1).

### Specificity and housekeeping enzyme

Specificity of the RT-PCR products was confirmed by a non-isotopic Southern blot hybridization procedure. A digoxigenin-labelled oligonucleotide probe was derived from the inner parts of amplified inner fragment. For hybridization and chemiluminiscent detection a commercially available kit (Lumigen PPD, Boehringer Mannheim, Germany) was used. The housekeeping enzyme glyc-

*© 2000 Cancer Research Campaign* 

**Table 1** Patients characteristics according to clinical stages of malignant melanoma

| Clinical stage (AJCC) | I | II | III | IV | All stages |
|---|---|---|---|---|---|
| Number of patients | 106 | 56 | 26 | 24 | 212 |
| Female sex | 61 (58%) | 33 (59%) | 16 (62%) | 12 (50%) | 122 (58%) |
| Site of the primary tumour | | | | | |
| Limb | 60 | 38 | 14 | 13 | 125 |
| TNS | 46 | 18 | 12 | 11 | 87 |
| Histological type of the primary tumour | | | | | |
| SSM | 80 | 15 | 6 | 6 | 107 |
| NM | 11 | 33 | 8 | 7 | 59 |
| ALM | 2 | 5 | 4 | 2 | 13 |
| LMM | 11 | 2 | 0 | 0 | 13 |
| Others | 2 | 1 | 8 | 9 | 20 |
| Age, median (range) | 53 (23–85) | 58 (20–85) | 57 (25–83) | 54 (23–83) | 55 (20–85) |

[a]TNS = trunk, neck, scalp and other primary tumour locations. [b]Others: ocular melanoma, mucosal melanoma or melanoma of unknown origin. SSM = superficial spreading melanoma, NM = nodular melanoma, ALM = acrolentiginous melanoma, LMM = lentigo maligna melanoma.

eraldehyde 3-phosphatase dehydrogenase (GAPDH) was used to test for integrity of the isolated RNA. Thirty PCR cycles were performed using the primers GAPDH 321 (ACCACAGTCCATGCCATCAC) and GAPDH 55 (TGCCAGCCCCAGCGTCAAAG). Only GAPDH-PCR-positive samples were evaluated, negative samples were considered to be non-informative.

### Additional control experiments

Sensitivity of the method was high enough to detect tyrosinase transcripts in 10 ml of control blood spiked with 10 SkMel28 cells (Figure 2). Additionally, blinded samples were successfully examined in the setting of the quality control initiative of the EORTC-Melanoma Cooperative Group (23).

### Statistics

Survival data were displayed as Kaplan–Meier estimates and compared between subgroups of patients using logrank tests. To assess the association between RT-PCR results and clinical stage, the Cochrane–Armitage trend test was used. To compare RT-PCR results with respect to patient's sex or site of the primary tumour, Mantel–Haenszel estimates of odds ratios and their 95% confidence intervals (CIs) were calculated, taking into account the stratification according to the clinical stages. All *P*-values are given two-sided. Multiple Cox regression was applied with backward elimination (significance level of 0.10) for selection of prognostic factors. The statistical analyses were performed with the Statistical Analysis System (SAS 1989) and StatXact (Version 3.0.2, 1996).

### RESULTS

A total of 212 blood samples were obtained from 212 melanoma patients with all clinical stages as they presented for routine tumour follow-up. Characteristics of included patients are displayed in Table 1, representing a profile of a typical follow-up cohort of melanoma patients. As expected, patients in early clinical stages were represented at a higher proportion, and likewise female sex was observed more frequently (57%). Furthermore, the proportion of different histologic melanoma subtypes and the sites of the primary tumours indicate that a typical follow-up cohort of melanoma patients was studied.

### Clinical stage, sex and site of the primary tumour – univariate statistical analysis

Overall, 46 of 212 blood samples (21.7%) were positive for tyrosinase RT-PCR. Positive results were obtained in 11% of stage I patients ($n$ = 106), 18% of stage II patients ($n$ = 56), 31% of stage III patients ($n$ = 26) and 67% of stage IV patients ($n$ = 24) as shown in Table 2, revealing a pronounced stage dependence ($P$ = 0.0001, Cochrane–Armitage). In addition, results were separately shown for females and males with respect to their clinical stage at time of blood sampling (Table 2). Remarkably enough, in all clinical stages, male patients showed a tendency towards higher rates of positivity for tyrosinase RT-PCR as opposed to female patients. The odds ratios listed in Table 2 demonstrate this fact. Overall, a positive RT-PCR was observed in 26 out of 90 male patients (28.9%) as compared to 20 out of 122 female patients (16.4%). The common odds ratio for a positive RT-PCR was 2.15 ($P < 0.05$) for males versus females, accounting for stratification for clinical stages (CI 1.05–4.40).

**Table 2** Clinical stage of patients positively tested by RT-PCR for tyrosinase discriminated for sex and the location of the primary tumour

| Clinical stage (AJCC) | I | II | III | IV | All stages | Statistical significance |
|---|---|---|---|---|---|---|
| Positive patients/all patients | 12/106 (11.3%) | 10/56 (17.9%) | 8/26 (30.8%) | 16/24 (66.6%) | 46/212 (21.7%) | $P < 0.0001$ |
| Positive test of males /total number of males. | 6/45 | 5/23 | 6/10 | 9/12 | 26/90 | $P < 0.05$ |
| Odds ratio male vs female | OR = 1.41 | OR = 1.56 | OR = 10.5 | OR = 2.14 | common OR = 2.15 | |
| Confidence interval | (0.42–4.70) | (0.39–6.14) | (1.50–73.7) | (0.38–12.2) | (1.05–4.40) | |
| Positive test of limb tumours /total number of limb tumours | 4/60 | 6/38 | 4/14 | 7/13 | 21/125 | $P < 0.05$ |
| Odds ratio limb vs not limb | OR = 0.34 | OR = 0.66 | OR = 0.80 | OR = 0.26 | common OR = 0.46 | |
| Confidence interval | (0.10–1.21) | (0.16–2.70) | (0.15–4.25) | (0.04–1.70) | (0.22–0.96) | |

OR = odds ratio.

© 2000 Cancer Research Campaign

**Table 3** Parameters affecting the survival intervals of 212 melanoma patients as selected by multiple Cox regression analysis. **A**. Disease-free survival. **B**. Overall survival

| | Hazard ratio | 95% confidence interval | *P*-values |
|---|---|---|---|
| **A** | | | |
| Clinical stage (AJCC) | | | |
| I or II (reference) | 1.0 | | |
| III or IV | 19.7 | (8.52–45.5) | *P* = 0.0001 |
| Positive tyrosinase RT-PCR in peripheral blood | 2.96 | (1.49–5.89) | *P* = 0.002 |
| **B** | | | |
| Clinical stage (AJCC) | | | |
| I or II (reference) | 1.0 | | |
| III or IV | 97.0 | (12.7–741) | *P* = 0.0001 |
| Positive tyrosinase RT-PCR in peripheral blood | 4.33 | (1.69–11.1) | *P* = 0.002 |

A similar observation was made comparing patients with primary melanomas on the limbs to patients with other primary tumour sites (Table 2). Patients with primary tumours on the limb showed a decreased risk to be tyrosinase positive in contrast to the other patients. Only 21 out of 125 melanoma patients with limb location of the primary tumour had positive test results (16.8%), while 25 out of 87 patients (28.7%) with other primary tumour sites had tyrosinase transcripts in their peripheral blood. The common odds ratio for clinical stages was 0.47 ($P < 0.05$) in favour of patients with primaries on the limb (CI 0.22–0.96). An association between any histological tumour type and a positive RT-PCR for tyrosinase was not detectable (data not shown).

### Association of positive RT-PCR assay and survival

During follow-up after PCR-blood testing, 23 of 25 melanoma relapses in stage I–III patients were detected by physical examination or ultrasound B-scan. Skin or regional soft tissues were affected with or without involvement of other organs. Only two stage III patients skipped the soft tissue compartment with tumour progression and became symptomatic from distant metastases. One patient developed headache 20 months after having been tested negative for blood-tyrosinase. Staging revealed multiple brain metastases causing death another 5 months later. The second patient showed dyspnoea 4 months after having been tested positive for blood-tyrosinase and died 3 months later from diffuse pulmonary metastases. However, CXR and abdominal ultrasound were negative at the time of blood sampling.

The median follow-up interval after blood sampling was 36 months for all patients (range 26–41 months). Disease-free survival was not evaluated for stage I or stage II patients, because only two and nine recurrences respectively, occurred in these groups. Disease-free intervals of patients with clinical stage III at time of blood sampling are displayed according to the method of Kaplan and Meier (Figure 1A), revealing a difference between patients with positive and negative tyrosinase RT-PCR ($P < 0.01$, log-rank). In the positively tested group only one patient is still without relapse since blood sampling for tyrosinase RT-PCR (disease-free interval: 27+ months) while the seven remaining patients meanwhile all had disease recurrences. Remarkably, in the negatively tested group only seven of 18 patients relapsed until to date.

Analysis of the overall survival interval from the time of blood sampling was only performed in stage IV patients (Figure 1B). Median survival of RT-PCR-positive patients was 8 months as compared to 12 months in the negative group, however, because of the limited number of patients this difference observed was not statistically significant ($P = 0.33$).

### Multiple regression analysis

To establish a model for prognosis of disease-free and overall survival, a multiple regression analysis with subsequent backward selection procedure was performed on all 212 patients including the following parameters: clinical stage (AJCC) of the patients (stage I or II versus stage III or IV), result of tyrosinase RT-PCR blood testing (positive versus negative), sex (female versus male), site of the primary tumour (limb location versus other locations) and histological subtype of the primary tumour (superficial spreading melanoma versus nodular melanoma versus others). Table 3 A and B show the results for relapse-free and overall survival. Out of these variables, clinical stage and the result of the tyrosinase RT-PCR are sufficient to give a prognosis on survival. All other parameters provide no additional information.

### DISCUSSION

Tyrosinase RT-PCR has been shown to facilitate the detection of melanoma cells in surgically removed lymph nodes (Wang et al, 1994), and in fine-needle aspirates as well (Schwürzer-Voit et al, 1996). In contrast, data published on tyrosinase RT-PCR of peripheral blood from melanoma patients are heterogeneous and sometimes inconclusive.

### Sensitivity of the tyrosinase RT-PCR

Generally, the sensitivity of control experiments has been described by most authors to be extremely high (Brossart et al, 1993; Kunter et al, 1996; Mellado et al, 1996; Reinhold et al, 1997; Ghossein et al, 1998) detecting one melanoma cell in 10 ml blood of healthy donors. However, analysis of blinded spiked samples revealed that such results might not be achieved routinely in all laboratories and a sensitivity of ten melanoma cells in 10 ml of blood is realistic (Keilholz et al, 1998). Detection rates for tyrosinase transcripts in peripheral blood of stage IV patients are described between zero (Foss et al, 1995) and 100% (Brossart et al, 1993) but are most likely due to technical differences. RNA extraction was performed from whole blood samples (Brossart et al, 1993; Foss et al, 1995; Kunter et al, 1996; Pitman et al, 1996; Van der Velde, 1996), from Ficoll separated fractions (Battayani et al, 1995; Hoon et al, 1995; Mellado et al, 1996; Reinhold et al, 1997; Ghossein et al, 1998) or following lysis of erythrocytes as described in the present study. RNA extraction was reported utilizing phenol precipitation of total RNA (Smith et al, 1991; Brossart et al, 1993; Hoon et al, 1995; Mellado et al, 1996; Jung et al, 1997; Reinhold et al, 1997) as well as following column purification of mRNA (Battayani et al, 1995; Van der Velde et al, 1996). Even data obtained by heterogeneous methods and by different PCR protocols, have been pooled within one report (Gläser et al, 1997).

### Specificity of tyrosinase RT-PCR

Mainly reports of very high sensitivities have raised doubts regarding the method's specificity and problems resulting from

© 2000 Cancer Research Campaign 

carry-over contamination have already been reported (Foss et al, 1995). However, positive tyrosinase RT-PCR results are usually not reported with regard to blood samples obtained from healthy control patients, from patients with benign diseases or from patients with non-melanoma diseases respectively (Brossart et al, 1993; Battyani et al, 1995; Hoon et al, 1995; Kunter et al, 1996). Besides examination of such control patients, testing of a typical set of follow-up patients with all clinical stages is helpful, since substantial carry-over contamination would affect all clinical stages in the same way and could thus be recognized.

### Univariate analysis – association with clinical stage, sex and primary tumour site

Despite large differences in sensitivity of tyrosinase RT-PCR, several authors reported a correlation of positive test results with clinical stages (Brossart et al, 1993, 1994; Battyani et al, 1995; Hoon et al, 1995; Kunter et al, 1996; Mellado et al, 1996; Farthmann et al, 1998) as we also observed (Table 2). In our study the rate of positive tests in stage IV patients was 67% (95% CI 44.6–84.4%), which is higher than the rates reported by many other authors. Most descriptions report rates below 30% for stage IV patients (Foss et al, 1995; Kunter et al, 1996; Pitman et al, 1996; Gläser et al, 1997; Jung et al, 1997) but also higher rates are described with 94% (Mellado et al, 1996) or even 100% (Brossart et al, 1993). In our study, the volume of peripheral blood examined was only 2.7 ml which is the lowest volume reported so far. Our finding of 11% and 18% positive patients in stage I and II respectively, corroborates already reported data between 10 and 25% for these stages (Brossart et al, 1993; Battyani et al, 1995; Mellado et al, 1996; Ghossein et al, 1998).

The question whether increased mortality rate of males (Balch et al, 1985) is reflected by tyrosinase RT-PCR so far has been addressed by only one study (Farthmann et al, 1998) finding a non-significant trend. Our study (Table 2) showed an odds ratio of OR = 2.15 for a positive RT-PCR in males ($P < 0.05$).

A very recent study (Farthmann et al, 1998) reported a significantly increased proportion of positive RT-PCR results in patients with nodular melanoma, with unknown primary tumour or with non-classifiable primary melanoma as compared to patients with other types. Our results (data not shown) did not confirm this finding.

Another known prognostic factor for survival, the site of the primary tumour, so far has not been evaluated in conjunction with tyrosinase RT-PCR in peripheral blood. We observed a decreased risk for positive RT-PCR in peripheral blood of patients with limb located primary tumours (OR = 0.46, $P < 0.05$) as compared to patients with other primary tumour sites (Table 2).

### Significance of positive tyrosinase RT-PCR for survival

Obviously, tyrosinase RT-PCR results obtained from analysis of peripheral blood samples, have to be reviewed upon their prognostic importance in the context of clinical follow-up of melanoma patients. So far, only few studies were able to address this topic on the basis of sufficiently long follow-up periods (Kunter, et al, 1996; Mellado et al, 1996, Ghossein et al, 1998). A significantly decreased disease-free interval was reported for 39 pooled stage II and stage III patients (Mellado et al, 1996). Recently, a significantly reduced overall survival interval was described in stage II patients as well as in stage III patients with tyrosinase RT-PCR positive assays (Ghossein et al, 1998). Concerning the overall survival of stage IV patients, a significant reduction was shown for tyrosinase-positive patients with visceral metastases (Kunter et al, 1996) while for the undivided cohort of stage IV patients such an association was not shown (Ghossein et al, 1998). Analysing blood samples, which were obtained from stage IV patients at first opportunity, no significantly different overall survival intervals were detected comparing tyrosinase RT-PCR positive and negative patients.

In stage III patients our tyrosinase RT-PCR results showed a strong association to disease-free survival. Within a median follow-up of 36 months after blood sampling all tyrosinase-positive patients relapsed, except one, while in the tyrosinase-negative group only seven of 18 patients had recurrences (Figure 1A). The clinical value of tyrosinase RT-PCR therefore should be greatest when testing this group of stage III patients.

### Multiple regression analysis

Frequently, statistical associations are reported without considering other important factors simultaneously. According to our multiple regression model on all 212 patients at least sex and primary tumour site were of no independent prognostic importance for disease-free and overall survival intervals. Both parameters had shown an association in univariate analysis with the detection of tyrosinase transcripts in peripheral blood. This is most likely the reason for the superior significance of RT-PCR results in multiple regression analysis. Sex and the site of the primary tumour are no longer significantly associated with survival intervals once RT-PCR is taken into account.

Therefore, clinical stage and the result of the blood tyrosinase RT-PCR comprised the whole information for prognosis in our study (Table 3). These results definitively underscore that multiple regression models are useful to identify valid prognostic factors.

### ACKNOWLEDGEMENTS

For expert help and discussions on technical procedures to Prof. J. Knop, Martina Willhauck and Ulrike Leiter.

### REFERENCES

Balch CM, Soong SW and Shaw HM (1985) An analysis of prognostic factors in 4000 patients with cutaneous melanoma. In: *Cutaneous Melanoma: Clinical Management and Treatment Results Worldwide*, Balch CM and Milton GW (eds), pp. 321–352. Lippincott: Philadelphia

Battyani Z, Grob JJ, Xerri L et al (1995) Polymerase chain reaction of circulating melanocytes as a prognostic marker in patients with melanoma. *Arch Dermatol* **131**: 443–447

Brossart P, Keilholz U, Willhauck M, et al (1993) Hematogenous spread of malignant melanoma cells in different stages of disease. *J Invest Dermatol* **101**: 887–889

Brossart P, Keilholz U, Scheibenbogen C, et al (1994) Detection of residual tumour cells in patients with malignant melanoma responding to immunotherapy. *J Immunother* **15**: 38–41

Farthmann B, Eberle J, Krasagakis K, Gstöttner M, Wang N, Bisson S and Orfanos CE (1998) RT-PCR for tyrosinase-mRNA-positive cells in peripheral blood: evaluation strategy and correlation with known prognostic markers in 123 melanoma patients. *J Invest Dermatol* **110**: 263–267

Foss AJE, Guille MJ, Occleston NL, et al (1995) The detection of melanoma cells in peripheral blood by reverse transcription-polymerase chain reaction. *Br J Cancer* **72**: 155–159

 *© 2000 Cancer Research Campaign*

Ghossein RA, Coit D, Brennan M, Zhang ZF, Wang Y, Bhattacharya, Houghton A and Rosai J (1998) Prognostic significance of peripheral blood and bone marrow tyrosinase messenger RNA in malignant melanoma. *Clin Cancer Res* **4**: 419–428

Gläser R, Rass K, Seiter S, et al (1997) Detection of ciculating melanoma cells by specific amplification of tyrosinase complementary DNA is not a reliable tumor marker in melanoma patients: a clinical two-center study. *J Clin Oncol* **15**: 2818–2825

Hoon DSB, Wang Y, Dale PS, et al (1995) Detection of occult melanoma cells in blood with a multiple-marker polymerase chain reaction assay. *J Clin Oncol* **13**: 2109–2115

Jung FA, Buzaid AC, Merrick IR, et al (1997) Evaluation of tyrosinase mRNA as a tumor marker in the blood of melanoma patients. *J Clin Oncol* **15**: 2826–2831

Keilholz U, Willhauck M, Rimoldi D, et al (1998) Reliability of reverse transcription-polymerase chain reaction (RT-PCR)-based assays for the detection of circulating tumor cells: a quality-assurance initiative of the EORTC Melanoma Cooperative Group. *Eur J Cancer* **34**: 750–753

Kirkwood JM, Strawderman MH, Ernstoff MS, Smith TJ, Bordern EC and Blum RH (1996) Interferon-alfa-2b adjuvant therapy of high risk resected cutaneous melanoma: the eastern cooperative oncology group trial EST 1684. *J Clin Oncol* **14**: 7–17

Kunter U, Buer J, Probst M, et al (1996) Peripheral blood tyrosinase messenger RNA detection in malignant melanoma. *J Natl Cancer Inst* **889**: 569–570

Kwon BS, Haq AK, Pomerantz SH, et al (1987) Isolation and sequence of a cDNA clone for human tyrosinase that maps at the mouse c-albino locus. *Proc Natl Acad Sci USA* **84**: 7473–7477

Mellado B, Colomer D, Castel T, et al (1996) Detection of circulating neoplastic cells by reverse transcriptase polymerase chain reaction in malignant melanoma: association with clinical stage and prognosis. *J Clin Oncol* **14**: 2091–2097

Pitman K, Burchill S, Smith B, et al (1996) Reverse transcriptase polymerase chain reaction for expression of tyrosinase to identify malignant melanoma cells in peripheral blood. *Annal Oncol* **7**: 197–201

Ponnazhagen S, Hou L and Kwon BS (1994) Structural organization of the human tyrosinase gene and sequence analysis and characterization of its promoter region. *J Invest Dermatol* **102**: 744–748

Proebstle T, Huber R, Sterry W (1996) Detection of early micrometastases in subcutaneous fat of primary malignant melanoma patients by identification of tyrosinase mRNA. *Eur J Cancer* **32A**: 1664–1667

Reinhold U, Lüdke-Handjery HC, Schnautz S, et al (1997) The analysis of tyrosinase-specific mRNA in blood samples of melanoma patients by RT-PCR is not a useful test for metastatic tumor progression. *J Invest Dermatol* **108**: 166–169

Schwürzer-Voit M, Proebstle TM and Sterry W (1996) Identification of lymph node metastases by use of PCR in melanoma patients. *Eur J Cancer* **32A**:264–268

Smith B, Selby P, Southgate J, et al (1991) Detection of melanoma cells in peripheral blood by means of reverse transcriptase and polymerase chain reaction. *Lancet* **338**: 1227–1229

Van der Velde, Zimmermann D, Roijers JF, Bouwens-Rombuds A, et al (1996) Molecular test for the detection of tumor cells in blood and sentinel nodes. *Am J Pathol* **149**: 759–764

Wang X, Heller R, VanVoorhis N, et al (1994) Detection of submicroscopic lymph node metastases with polymerase chain reaction in patients with malignant melanoma. *Ann Surg* **220**: 768–774

© 2000 Cancer Research Campaign
